# Dynamic Changes in Protein Functional Linkage Networks Revealed by Integration with Gene Expression Data

**DOI:** 10.1371/journal.pcbi.1000237

**Published:** 2008-11-28

**Authors:** Shubhada R. Hegde, Palanisamy Manimaran, Shekhar C. Mande

**Affiliations:** Centre for DNA Fingerprinting and Diagnostics, Nacharam, Hyderabad, India; Peking University, China

## Abstract

Response of cells to changing environmental conditions is governed by the dynamics of intricate biomolecular interactions. It may be reasonable to assume, proteins being the dominant macromolecules that carry out routine cellular functions, that understanding the dynamics of protein∶protein interactions might yield useful insights into the cellular responses. The large-scale protein interaction data sets are, however, unable to capture the changes in the profile of protein∶protein interactions. In order to understand how these interactions change dynamically, we have constructed conditional protein linkages for *Escherichia coli* by integrating functional linkages and gene expression information. As a case study, we have chosen to analyze UV exposure in wild-type and SOS deficient *E. coli* at 20 minutes post irradiation. The conditional networks exhibit similar topological properties. Although the global topological properties of the networks are similar, many subtle local changes are observed, which are suggestive of the cellular response to the perturbations. Some such changes correspond to differences in the path lengths among the nodes of carbohydrate metabolism correlating with its loss in efficiency in the UV treated cells. Similarly, expression of hubs under unique conditions reflects the importance of these genes. Various centrality measures applied to the networks indicate increased importance for replication, repair, and other stress proteins for the cells under UV treatment, as anticipated. We thus propose a novel approach for studying an organism at the systems level by integrating genome-wide functional linkages and the gene expression data.

## Author Summary

Many cellular processes and the response of cells to environmental cues are determined by the intricate protein∶protein interactions. These cellular protein interactions can be represented in the form of a graph, where the nodes represent the proteins and the edges signify the interactions between them. However, the available protein functional linkage maps do not incorporate the dynamics of gene expression and thus do not portray the dynamics of true protein∶protein interactions in vivo. We have used gene expression data as well as the available protein functional interaction information for *Escherichia coli* to build the protein interaction networks for expressed genes in a given condition. These networks, named conditional networks, capture the differences in the protein interaction networks and hence the cell physiology. Thus, by exploring the dynamics of protein interaction profiles, we hope to understand the response of cells to environmental changes.

## Introduction

Gene expression pattern in all organisms is a property of the environmental conditions in which they grow. Expression of a large number of genes is turned on or off conditionally and temporally allowing the organisms to adapt to different growth or changing environmental conditions. While some genes are constitutively expressed under many different conditions, presumably being essential for the organism to carry out basic cellular processes for growth and sustenance, many genes are expressed only under defined conditions. DNA microarray offers a powerful tool to study such gene expression profiling. Studying the gene expression pattern under different conditions therefore offers an attractive approach to study the response of an organism to changing environmental conditions.

The traditional analysis of microarray data involves measuring differential expression between two samples after background elimination and data normalization. An unsupervised classification method such as clustering or principal component analysis is popularly used to identify genes that have a similar regulation pattern [1],[2]. Although measuring relative gene expression levels is the preferred method of analysis, the individual signal intensities, which contain valuable information on absolute gene expression, are often not considered. Studying the pattern of absolute expression of genes, rather than relative expression between two conditions, might provide an alternate useful approach for comparative analysis.

A few attempts have been made to analyze differences in gene expression arising out of different conditions of growth. The gene expression profiling in *E. coli* has revealed varied mRNA transcripts in the cells growing in minimal and rich media as well as in their exponential and transitional phases of growth [3]. In a similar line, the gene expression dynamics and its relevance to *E. coli* physiology is shown by the protein expression profiles and their correlation with the gene expression profiles [4],[5]. Absolute gene expression analysis in fission yeast has shown that the basic cellular functions are carried out by the conserved genes and that the organism specific genes are expressed conditionally for the specialized processes [6]. These studies have provided a wealth of data on the molecular and genetic basis of response of organisms to changing environmental conditions.

While the analysis of gene expression data provides useful insights into the adaptation process, it is believed that the response of organisms is dictated by the dynamics of biomolecular interactions profile. One of the inspirations to carry out the present study was to understand the changing landscape of protein-protein interactions under different environmental conditions. The protein-protein interaction studies carried out experimentally usually represent only a fraction of all the possible interactions among different cellular proteins [7]. Moreover, the protein interaction networks available, for example in *E. coli* [8],[9], represent only the static protein interaction networks and are able to capture the interactions involving only those genes whose products are expressed under the unique experimental conditions. On the other hand, understanding of the dynamics of protein interaction networks demands profiling genome-wide protein-protein interactions under many different experimental conditions. Such experiments are currently prohibitive in time and resources. Therefore, profiling interactions under changing environmental conditions presents enormous challenge to the experimental biologists.

There have been a few attempts to combine protein: protein interaction networks and gene expression data [10]. The dynamics of yeast interactome studied using mRNA expression revealed two kinds of hubs namely, “date hubs” and “party hubs” [11]. The former are believed to bind different proteins at different time or location, and the latter are believed to bind to their partners simultaneously. Also, the hub proteins were shown to have lower levels of differential expression compared to the non-hub proteins [12]. Further, it was observed that static and dynamic proteins cluster into different modules in yeast protein interaction network [13]. In another study the PPI networks were studied in the context of genes expressed during aging [14]. These sub-networks and the modules therein were examined for understanding the aging process. Thus, although a few attempts have been made in integrating the gene expression data with protein interaction networks, the analysis of differential gene expression in the context of corresponding networks remains an underexplored area. Our study is an attempt in this direction.

## Results/Discussion

We have used gene expression information of *E. coli* to identify the genes that are expressed in the prevailing conditions and constructed reduced networks from the predicted genome-wide parent functional linkage network. Figure S1A schematically shows our approach, which we intend to use for the analysis of *E. coli* expression data. The sub-networks thus constructed are hypothesized to represent a real functional interaction picture of the cell. We have further applied various graph theoretical measures to extract the relevant biological information from these sub-networks. This we propose to be a novel methodology in which a raw microarray data can be analyzed by incorporating molecular interaction information with gene expression.

### Construction of the Conditional Protein–Protein Interaction Networks

Predicted functional interaction network for *E. coli*, which comprises 78,048 interactions among 3,682 proteins, was used as the parent network [15]. This functional linkages network was obtained by training a Support Vector Machine on high confidence interactions in the EcoCyc database and assuming that cytoplasmic and periplasmic protein do not interact with each other. The predicted data set has fewer interactions in common with the experimentally derived networks [8],[9]. However, the overlap increases significantly if the indirect interactions are taken into consideration.

As a case study, we have chosen to study the gene expression data from UV exposure in wild type and SOS deficient *E. coli* at 20 minutes post irradiation [16]. The four conditions studied are Untreated Wild Type (UWT), UV treated Wild Type (TWT), untreated *lexA* mutant (UML) and UV treated *lexA* mutant (TML). Graph theoretical measures were applied to the four sub-networks that were derived by this methodology, and then compared amongst each other (Figure S1B).

The *E. coli* genome has the capability to encode more than 4000 genes. Out of these, approximately 40–60% are likely to be expressed under any defined condition [4]. Using the methodology described in Materials and Methods, our processing of the raw microarray data revealed expression of around 2000 genes (Table S1), which is in close agreement with the earlier studies on absolute gene expression [5]. Networks under each of the four conditions (henceforth referred to as conditional networks) were constructed by mapping the expressed proteins on the parent network. The conditional networks possess around 30,000 interactions among the expressed proteins.

### Global Properties of the Conditional Networks

It is anticipated that the effect of turning off or on of the genes expressed under the four conditions will be reflected in the conditional networks. While, this is likely to lead to many local perturbations in the network, the global properties of the four networks are not likely to change significantly. Various topological properties of the conditional networks under the perturbations such as mutation (*lexA*) or UV treatment along with the network corresponding to wild-type were therefore studied.

The four conditional networks exhibit similar network parameters (Table 1). The core cluster comprises >95% of the nodes in the network. The overlap of the interactions and the nodes in UWT-TWT and UML-TML is shown in Figure S2. The degree distributions in all the four conditional networks show power law behavior with the degree exponent of 1.1. Therefore, the networks are scale-free, indicating that these are similar to other real world networks. The scale-free property also is suggestive of their resistance to random node failure [17]. The network diameters for the studied graphs imply the small-world property where the number of steps required for reaching from one node to the other is not more than 9. Other properties such as average clustering coefficient and mean eccentricity are similar in all the graphs (Table 1). The fractal dimensions calculated using cluster growing method indicates that the networks are self similar with all the four conditional networks possessing similar fractal dimensions. Similarly, network efficiency is also comparable in the conditional networks in spite of the imposed perturbations. Thus, we do not find any significant difference in the global network properties of the four conditional networks.

**Table 1:** Global properties of the sub-networks.

Property	Parent Network	UWT	TWT	UML	TML
Nodes	3,682	1,899	1,865	1,957	1,947
Edges	78,048	34,893	34,680	31,900	33,513
Percentage core nodes	96.9	97.4	97.9	96.1	95.5
Average degree	42.4	36.7	37.2	32.6	34.4
Degree exponent	1.2	1.1	1.1	1.1	1.1
Diameter	11	8	8	8	9
Mean eccentricity	7.99	5.66	5.78	5.89	6.07
Average clustering coefficient	0.23	0.21	0.21	0.22	0.22
Fractal dimension	3.9	3.5	3.4	3.5	3.5
Network efficiency	0.36	0.37	0.38	0.36	0.37

Global network parameters for the parent network (15) and the conditional networks. UWT, UV Untreated Wild Type; TWT, UV Treated Wild Type; UML, UV Untreated *lexA* mutant; TML, UV Treated *lexA* mutant.

### Unique Nodes of the Conditional Networks

Each of the four conditional networks possesses unique nodes corresponding to the genes that are expressed differentially. Interestingly, the uniquely expressed genes include a few hubs and transcription factors. The lists of proteins that are identified to be uniquely expressed are listed in the Table S2. As anticipated, some of the proteins involved in DNA repair, recombination and cell structure determination are observed only in the UV treated wild type network in comparison with the untreated wild type network. In addition to the UV damage response genes, other environmental stress related genes are also uniquely expressed in the UV treated wild type cells. Thus, the comparative analysis of PPI networks appears to identify a few crucial features that might be physiologically relevant.

Mapping of the unique nodes of each of the four comparison sets to different metabolic pathways for *E. coli*, as enlisted in KEGG database [18], revealed that replication and repair proteins, as expected, are more in number in the UV treated networks compared to their untreated counterparts (Figure S3). Interestingly, many genes coded on carbohydrate metabolism operons are seen only in the untreated wild type network. The presence of these gene clusters in the untreated wild type cells and their absence in UV irradiated cells appears to suggest the repression of sugar metabolism in UV treated *E. coli* cells. Earlier study has shown that a few carbohydrate metabolism operons indeed exhibit reduced expression under UV exposure [16]. Similarly, we observe genes belonging to membrane transport more in number as uniquely expressed in the *lexA* mutant cells when compared with the respective wild type cells. Thus, the four-way comparisons of expressed genes, as anticipated, highlight the importance of DNA repair and replication processes under UV exposure.

One of the interesting genes that we observed to be expressed only in the UV treated cells is the *hda* gene, protein product of which is involved in DnaA inactivation. It has been demonstrated earlier that cells suppress replication upon DNA damage. As Hda is known to repress hyper initiation of DNA replication by inactivating DnaA [19], the criticality for Hda in the UV treated networks appears to be significant. Interestingly, this protein has a high degree in the UV treated networks (Table S3). Since high degree nodes are believed to be important in maintaining robustness of graphs [17], the high degree of Hda and its unique expression in UV treated cells signifies its importance when the cells are treated with UV radiation. Furthermore, this gene is observed to be expressed under UV exposure, both in the wild type as well as in the *lexA* mutant, suggesting that its expression is independent of the well characterized SOS response.

Another interesting example is the unique expression of genes involved in the iron uptake system in the untreated wild type cells. The proteins EntA, EntB and EntF function in the pathway of enterobactin synthesis and the proteins FepA and FepB form a part of the channel to transport Fe-enterobactin complex inside the cell. When cells are UV treated, reactive oxygen species (ROSs) are synthesized via photo-Fenton reaction which leads to oxidative damage of structural proteins, enzymes, DNA and lipids. Thus, it is likely that cells repress iron uptake to protect cellular macromolecules from damage. The absence of these iron uptake proteins from UV treated wild type network supports this idea.

The analysis of uniquely expressed nodes under one condition, but not in another condition, indicates some of the possible effects of UV radiation on *E. coli*. The importance of repression of carbohydrate metabolism and iron uptake upon exposure to UV is apparent in the networks. Similarly, an important hub, Hda, is also apparently expressed only upon UV exposure. Thus, the uniquely expressed nodes in the networks indicate of how *E. coli* might respond to UV, thereby suggesting that such an analysis might be useful in other similar studies.

### Analysis of the Path Length Differences

An interesting aspect in systems analysis is to study the effect of selective removal of nodes on modifications in the shortest path lengths in the conditional networks. The shortest path lengths in a network signify the efficiency of communication between the nodes, and any alteration in these paths might suggest significance of these nodes under the two conditions. Importantly, the overall diameter of the four conditional networks is identical, indicating that diameter as a global property of the network is not subject to change. Moreover, all the networks have small world property; almost all nodes can be reached from every other in a small number of steps. This is not surprising, considering the biological robustness that is reflected in these networks. Thus, analysis of shortest path lengths might yield interesting insights into the relative importance of communication networks in the four sub-networks.

In order to analyze local changes in pathlength differences, the reduced pathlength matrices were constructed for the common nodes in network pairs under study. The pathlength difference of more than or equal to 3 for each node pair in two reduced networks were considered significant. As expected, for most of the node pairs, there is no change in the pathlength as there are multiple paths to reach from one node to another node even in the event of a collapse of a particular path. Interestingly, we observe considerable variation in the path for some node pairs, manifesting their reduced connectivity in terms of efficient information exchange, two examples of which are discussed in detail below.

**Figure 1:**
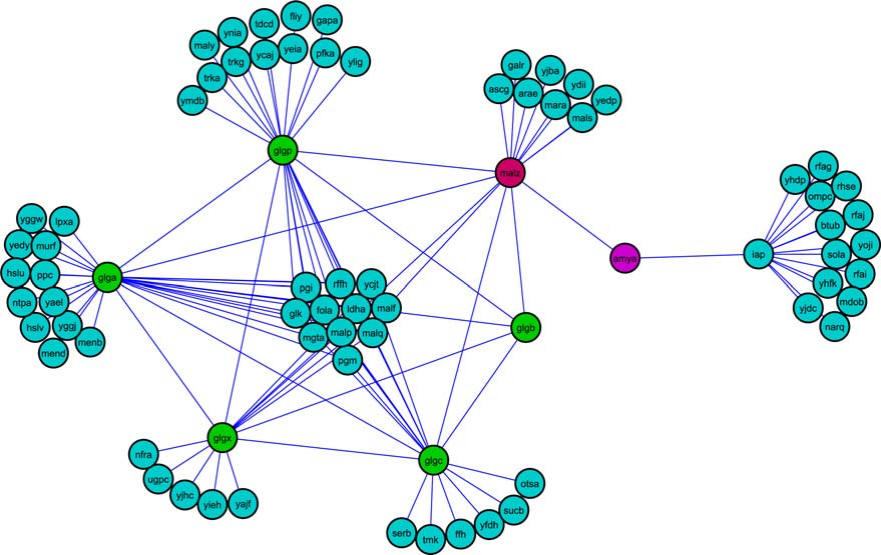
Path length analysis for the starch and sugar metabolism genes. In the untreated wild type network, the subgraph for starch and sucrose metabolism pathway proteins is well connected. The absence of MalZ in treated wild type network increases the path from AmyA to some of the glycogen metabolism proteins significantly.

The shortest pathlength from AmyA, a cytoplasmic α-amylase to many of the glycogen metabolism enzymes is observed to be increased in UV treated wild type network (Figure 1). In the untreated wild type subgraph, AmyA is connected to glycogen metabolism enzymes through MalZ, which functions as a maltodextrin glucosidase. The increase in the path length is due to the absence of MalZ node in the cells treated with UV and thereby resulting in isolation of AmyA with respect to proteins belonging to starch and sucrose metabolism in *E. coli*. Thus, the importance of repression of carbohydrate metabolism upon UV treatment is highlighted not only by the repression of a few carbohydrate metabolism operons [16], but also by the reduced efficiency of communication between different glycogen metabolism genes.

**Figure 2:**
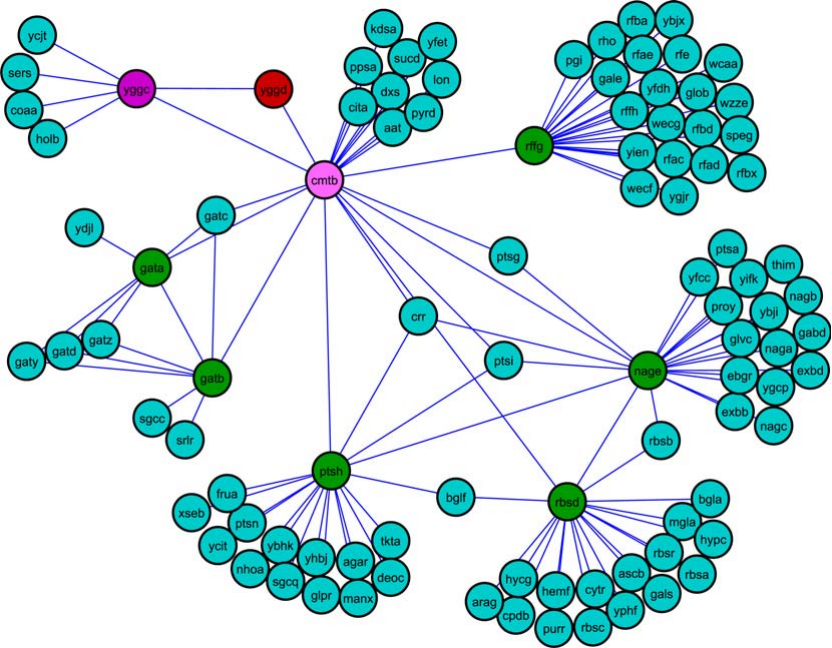
Path length analysis for the nodes of phosphotransferase system. The absence of CmtB in the UV treated *lexA* mutant network increases the pathlength from YggD, a putative *cmt* operon transcriptional regulator to other phospho transferase system (PTS) enzymes.

Another interesting example pertains to the phosphotransferase system in *E. coli*. The sub-network corresponding to a part of phosphotransferase (PTS) system in *E. coli* in the untreated mutant network is very well connected, whereas the mutant UV treated network lacks CmtB, a component of mannitol PTS permease (Figure 2). This results in the increased shortest paths from YggD, which is a hypothetical transcriptional regulator of *cmt* operon, to other proteins which are part of the phosphotransferase system in *E. coli*. Since PTS regulates the uptake and metabolism of several sugars, we speculate that the increased paths between YggD and other PTS components might be a strong indication for the temporal repression of sugar metabolism in cells treated with UV radiation.

### Expression of the Hubs

It has been reported that the highly connected nodes of the network (hubs) are three times more likely to be essential than the poorly connected nodes [20]. Moreover, dynamics of yeast interactome has revealed two kinds of hubs, ones which are present under a variety of conditions, and the ones which appear only under certain specific conditions [11]. Thus, it is important to analyze the presence or absence of hubs under the four conditions. We have defined hubs of the parent network as the nodes having degree more than 60 and thereby identified 736 hubs in the network. We find that around 70–75% of the hubs of the parental network are expressed in the conditional networks. As a null hypothesis, when test networks are constructed by choosing random nodes of the parent network, we find only 50% hubs (Figure S4). Thus, essentiality of the hubs appears to manifest itself by the expression of a large number of hubs under all the four different conditions. One such example of the Hda hub was described above.

### Centrality Measures

It is likely that the importance of a functional role of a gene might differ according to the prevailing condition of growth. The relative importance of a node in graph theory can be assessed by calculating various centrality measures. We have therefore analyzed different centrality measures of graph theory with respect to their relevance to the four sub-networks.

Degree centrality is based on how well the node is connected in a graph. Degree centrality thus states that a node tends to be essential in a network if it is highly connected and its removal has severe impact on the overall topology and connectedness of the network [17],[20]. Similarly, if a node is positioned in such a way that it can communicate with other nodes quickly then the node is considered to be important in terms of closeness centrality. Betweenness centrality, on the other hand measures the number of shortest paths that traverse through a node. Both closeness and betweenness centrality have also been reported to be good measures to assess gene essentiality [21]–[23]. Based on these observations, we calculated the three centrality values for the nodes in the conditional networks, and then for each of the nodes we computed the pair wise difference between the different conditions.

To address conditional or relative criticality of a node, we calculated the difference in the centrality measures for the common nodes in the comparison set. The centrality measure difference is approximately normally distributed, thus about 99.7% values are expected to lie within 3 standard deviations of the mean value. For most of the nodes, there is no change in the centrality value as expected. We have chosen to study those proteins whose centrality measure difference is more than 3 times the standard deviation of the distribution. When untreated wild type and UV treated wild type networks are compared, the proteins belonging to carbohydrate metabolism and energy metabolism such as BglX, Dld, GatB, GlgA, CydA, CydB and YneH have greater centrality measure in the untreated wild type network. The replication and repair proteins, namely RecN, RecO, Tag, HepA and HolC on the other hand have greater centrality values in the UV treated wild type network. Likewise, DnaA, DnaE, Mfd, RecJ and SbcB functioning in the replication and repair machinery possess significantly higher centrality values in UV treated mutant networks compared to their untreated counterparts. We observe no considerable change in the centrality measure for the proteins of the pathways such as polyketide biosynthesis, cell motility and xenobiotics biodegradation. A detailed list of proteins with significant difference in centrality along with their functions in UWT- TWT and UML- TML comparison set is given as Table S4. In order to check if the standard deviation cutoff (3.0) has any effect on our overall conclusions, we reanalyzed the data on the centrality using a cutoff of 2.0. However, the overall conclusions on the importance of carbohydrate metabolism in untreated cells, and those of DNA replication and repair in the UV-treated cells, remain the same. We may thus conclude that the cutoff value of standard deviation, if modified, does not appear to alter the overall biological conclusions.

**Figure 3:**
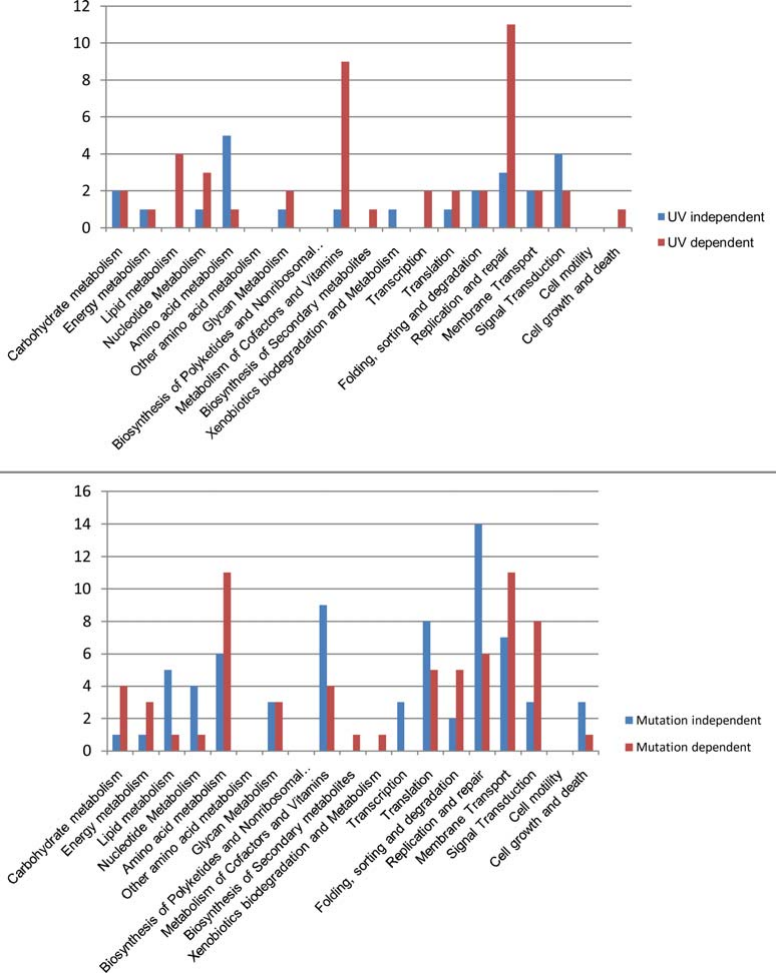
Classification of hubs according to the KEGG metabolic pathways. The high degree nodes of each of the four conditions are classified as critical in a UV treatment dependent or independent manner and *lexA* mutation dependent or independent manner, and they are mapped onto different metabolic pathways of *E. coli* as enlisted in the KEGG database.

Further, to study the essentiality of the nodes depending on the UV treatment or the *lexA* mutation, we undertook degree centrality analysis of top 30% nodes in each of the conditional networks. Using these criterion more than 550 nodes can be classified as high degree nodes under each condition (Table 2). When the common high degree nodes of wild-type networks (untreated as well as UV treated) and the common high degree nodes of *lexA* mutant (untreated as well as UV treated) networks are compared, interestingly 104 high degree nodes are unique to the wild type networks but are absent in either one or both mutant networks. Similarly 100 high degree nodes are unique to the mutant networks but are absent in either one or both wild type networks. We therefore consider them as the nodes that are essential in a mutation independent and mutation dependent manner respectively. A similar comparison for the high degree nodes in the context of UV treatment revealed 42 nodes as essential depending on UV treatment and 57 nodes as essential when there is no UV treatment (Table 2). We further mapped these proteins onto different metabolic pathways in the KEGG database and classified them as *lexA* mutation independent, *lexA* mutation dependent, UV treatment dependent and UV treatment independent (Figure 3 and Table S5). Some specific examples of this analysis are discussed below.

**Table 2:** Degree centrality of the conditional networks.

Conditional Networks	Number of High Degree Nodes	Networks Compared	Common Nodes	Criticality	Number of Proteins
UWT	570	UWT-TWT	527	Mutation independent	104
TWT	560	UML-TML	523	Mutation dependent	100
UML	587	UWT-UML	465	UV independent	42
TML	584	TWT-TML	480	UV dependent	57

The analysis of the top 30% nodes in terms of degree centrality alone in each conditional network revealed the nodes that are proposed to be essential depending on the UV treatment or the mutation. UWT, UV Untreated Wild Type; TWT, UV Treated Wild Type; UML, UV Untreated *lexA* mutant; TML, UV Treated *lexA* mutant.

We are able to identify many repair proteins such as DinG, DnaN, MutM, MutS, RuvC, Rep and RecF that are likely to be indispensable for the UV treated networks in terms of degree centrality. The criticality of some of the proteins that belong to lipid metabolism and cofactors and vitamins metabolism seems to be UV treatment dependent. One of the proteins that appears to be important from our anslysis in UV treated cells is UspA, the universal stress protein. Earlier study has shown the role of UspA in resistance to DNA damaging agents and that its regulation is *lexA* independent [24]. The mutants lacking *uspA* were shown to be sensitive to UV irradiation. It is interesting to observe the importance of this protein through network centrality studies, which was otherwise not obvious from the classical microarray analysis. Similarly, our analysis also suggests the importance of the protein, ApaH. ApaH functions as a diadenosine tetraphosphatase and its substrate AppppA has been reported to regulate cell division [25], affect cell motility and catabolite repression [26] and shown to bind to several heat shock and oxidative stress proteins [27]. Our observation that ApaH is critical in the studied networks in an UV dependent manner seems to be relevant in this regard.

The analysis carried out by us is based on the predicted genome-wide functional linkages [15]. In order to examine if the properties observed for the conditional sub-networks are consistent with those obtained from experimentally validated protein∶protein interactions, we further carried out similar analysis for the two available experimental protein∶protein interaction networks [8],[9]. The core protein interactions in the network reported by Butland *et al.* covers 1,255 proteins and 5,395 interactions among them [8]. The average degree for the core network is approximately 8.5 and the clustering coefficient is ∼0.085. Similarly, the core protein interactions in the network reported by Arifuzzaman *et al.* covers 2,927 proteins and 11,105 interactions [9]. The average degree for the core network is approximately 8.5 and the clustering coefficient is ∼0.065. The low average degree and clustering coefficient for the experimental networks compared to the predicted functional linkages might be due to the inability of experimental methods to saturate the genome-wide interaction networks.

The obtained conditional networks derived from the experimental interactions show topological robustness similar to their parent networks. Interestingly, similar to the conclusions that we have drawn based on the analyses derived from functional linkages network, we observe the UV-dependent criticality of many of the replication and repair proteins through network centrality analysis as well as the analysis of unique nodes of the networks. We also observe the expression of ∼65% hubs in conditional networks derived from Arifuzzaman *et al.* data [9] and ∼85% hubs in the ones obtained from Butland *et al.* data [8]. These numbers being significantly higher than those by choosing nodes in the networks randomly, suggest that one may obtain biologically important insights through such an analysis.

Some of the cutoffs applied in our study might appear to be superficially arbitrary. For example, a gene was considered to be expressed if the net signal intensity corresponding to its spot was more than or equal to the median signal intensity of the spots within the sector. Although this cutoff might seem arbitrary, the rationale for using median was based on the observation that gene expression is a stochastic event and hence the expression of a gene as well as copy number of the expressed protein differs from cell to cell even in an isogenic cell population [28],[29]. Despite the inherent stochasticity, the response of a colony of bacteria to external stimuli is based on simultaneous expression of a set of genes. It is reported that the noise in gene expression is inversely proportional to the mean expression level [30] and also that essential genes have lower noise in their expression [31]. Therefore, with median as our cutoff, we can eliminate the noisy expression and identify those as genes as expressed which have: (i) high expression levels and (ii) respond to the growth condition. In order to minimize noise in such an identification process we have considered each sector of the chip independently to overcome the differences in the environments within the chip that contribute to expression variances. Thus, with our cutoff of 1.0, we see a reasonable of number of genes being expressed, i.e. ∼40–60% [4], and also expression of approximately 75% of hub proteins.

We further tested the effect of different cutoffs on the overall conclusions of our analysis. With the cutoff of 0.9 and 1.1, we observe that our earlier conclusions, such as increased importance of replication and repair proteins, and cofactor metabolism proteins in the UV treated cells, repression of carbohydrate metabolism upon UV treatment and importance of unique nodes of the conditional networks, remain identical. With further modification of these cutoff values to 1.2, we observe approximately 1600 genes being expressed which might be considered fewer than anticipated [4]. Similarly with modification of the cutoff value to 0.8, many more proteins are considered to be expressed, which might lead to noise in the expression analysis. Thus, although the cutoff value of 1.0 appears arbitrary, it leads to reasonable hypothesis on the response of *E. coli* to UV.

Thus, the comparative analysis does indeed reveal physiologically important changes in the four networks. Some of these changes would not have been apparent by measuring gene expression alone, or by the standard analysis of microarray data. This is partly due to the fact that the levels of expression of many genes do not change under different conditions, but nonetheless the profile of interactions surrounding them changes significantly, thereby altering their significance in the broader picture of the cell. In this manner, studying the dynamics of protein∶protein interactions appears to hold promise for the systems level understanding of an organism.

The analysis proposed in this study can also be potentially applied to disease interaction networks. For example, understanding how the interactions within a pathogen or a host change during the disease process, and the implications of these changes might yield useful insights into the disease. Further, this information can be used to deriving novel therapies against the diseases.

## Methods

### Microarray Data Processing and PPI Network Construction

The raw microarray data for *E. coli* were downloaded from the Stanford Microarray Database (SMD, http://smd.stanford.edu/) [32]. The SMD lists sector information of the chip, and it is likely that environments within the chip differ considerably. Each spot was therefore assigned to one of sixteen possible sectors in the chip using sector information given in the raw data. A gene was considered to be expressed if the net signal intensity (i.e. background corrected) corresponding to its spot was more than or equal to the median signal intensity of the spots within the sector.

The conditional protein interaction network was built for the expressed genes by mapping them onto an existing predicted functional interaction network for *E. coli* [15]. All orphan nodes were removed from the obtained network and the core interaction network was used for further analysis.

### Global Properties of the Network

Network properties such as average degree, degree exponent, diameter, average clustering coefficient were calculated according to [33]. The mean eccentricity was calculated according to [34]. Fractal dimension was measured using cluster growing method [35]. Network efficiency is the property that quantifies how well the nodes of the network exchange information, and this parameter was calculated according to [36].

### Path Length Analysis

The shortest paths for all pairs of nodes in the network were calculated by Dijkstra's algorithm [37]. The difference in the path for a node pair in two different networks was analyzed.

### Centrality Measures

Network centrality measures like degree centrality, closeness centrality and betweenness centrality were calculated [36]. The definitions of these centrality measures are as follows:

Degree centrality of a node *i* in the network G is
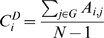
where, *A_i,j_* is the element in the adjacency matrix A for the nodes *i* and *j*, and N is the total number of nodes in G.

Closeness centrality of a node *i* in the network G is
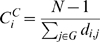
where *d_i,j_* is the shortest path between *i* and *j*.

Betweenness centrality of a node *i* in the network is
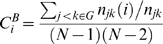
where, *n_jk_*(*i*) is the number of shortest paths between *j* and *k* that traverse through *i* and *n_jk_* is the total number of shortest paths between *j* and *k*.

### Sub-Network Visualization

Sub-networks were visualized and analyzed using Cytoscape 2.4.1 [38] and NAViGaTOR (http://ophid.utoronto.ca/navigator/).

## Supporting Information

Table S1 Microarray data processing and network information for the conditional networks.
(0.05 MB RTF)

Table S2 Uniquely expressed genes list: Four-way comparison.
(0.09 MB XLS)

Table S3 Interacting partners of Hda in the UV treated wild-type network with their functions, classified according to functional classes.
(0.06 MB RTF)

Table S4 Functions of the high centrality measure nodes in the comparison set UWT-TWT and UML-TML.
(0.07 MB RTF)

Table S5 List of mutation dependent/independent and UV treatment dependent/independent proteins
(0.03 MB XLS)

Figure S1 Differential gene expression and comparison of networks. (A) Pictorial representation of differential gene expression in the network context. Red and green represent the nodes expressed uniquely under the defined conditions, whereas blue nodes are expressed under both the conditions. (B) Four-way comparison of the networks. UWT, wild type; TWT, UV treated wild type; UML, lexA mutant; TML, UV-treated lexA mutant.
(0.47 MB TIF)

Figure S2 The overlap of the interactions and the nodes in UWT-TWT and UML-TML.
(0.76 MB TIF)

Figure S3 Mapping of unique nodes to different metabolic pathways.
(0.45 MB TIF)

Figure S4 Expression of the hubs.
(0.23 MB TIF)
