# Supplementary material for: Dynamic Changes in Protein Functional Linkage Networks Revealed by Integration with Gene Expression Data
Source: PLoS Comput Biol. 2008 Nov 28;4(11):e1000237. doi: 10.1371/journal.pcbi.1000237 (PMC2580820; doi:10.1371/journal.pcbi.1000237)
Supplement: Table S1 — Microarray data processing and network information for the conditional networks. (0.05 MB RTF) [file pcbi.1000237.s001.rtf]

Supplementary Table 1: Microarray data processing and Network information for the conditional networks.
Data Type	Untreated wild type	UV Treated wild type	Untreated mutant	UV treated mutant	
Data Set	1278, Ch2	1282, Ch2	1908, Ch2	1912, Ch2	
Number of genes expressed	2,405	2,398	2,627	2,621	
Number of common genes in the parent network	1,949	1,905	2,037	2,039	
Number of Interactions	34,905	34,684	31,922	33,543	
Number of core interactions	34,893	34,680	31,900	33,513	
Number of core unique nodes	1,899	1,865	1,957	1,947	
 
 
