# Supplementary material for: Dynamic Changes in Protein Functional Linkage Networks Revealed by Integration with Gene Expression Data
Source: PLoS Comput Biol. 2008 Nov 28;4(11):e1000237. doi: 10.1371/journal.pcbi.1000237 (PMC2580820; doi:10.1371/journal.pcbi.1000237)
Supplement: Table S3 — Interacting partners of Hda in the UV treated wild-type network with their functions, classified according to functional classes. (0.06 MB RTF) [file pcbi.1000237.s003.rtf]

Supplementary Table S3:

Interacting partners of Hda in the UV treated wild-type network with their functions, classified according to functional classes.

Lipid Metabolism
cdsA	: CDP-diglyceride synthase
fabD	: malonyl-CoA-[acyl-carrier-protein] transacylase

Nucleotide Metabolism
dnaQ	: DNA polymerase III epsilon subunit
dnaX	: DNA polymerase III/DNA elongation factor III, tau and gamma subunits
holA	: DNA polymerase III, delta subunit
holB	: DNA polymerase III, delta prime subunit
ppx	: exopolyphosphatase
relA	: (p)ppGpp synthetase I/GTP pyrophosphokinase

Amino acid Metabolism
murE	: UDP-N-acetylmuramoyl-L-alanyl-D-glutamate:meso-diaminopimelate ligase
murF	: UDP-N-acetylmuramoyl-tripeptide:D-alanyl-D-alanine ligase
pheT	: phenylalanine tRNA synthetase, beta subunit
proS	: prolyl-tRNA synthetase

Other amino acid Metabolism
murD	: UDP-N-acetylmuramoyl-L-alanine:D-glutamate ligase

Glycan Metabolism
amiC	: N-acetylmuramoyl-L-alanine amidase
ftsI	: transpeptidase involved in septal peptidoglycan synthesis (penicillin-binding protein 3)
murD	: UDP-N-acetylmuramoyl-L-alanine:D-glutamate ligase
murE	: UDP-N-acetylmuramoyl-L-alanyl-D-glutamate:meso-diaminopimelate ligase
murF	: UDP-N-acetylmuramoyl-tripeptide:D-alanyl-D-alanine ligase
murG	: N-acetylglucosaminyl transferase

Cofactors and Vitamins Metabolism
bioD	: dethiobiotin synthetase

Transcription
nusA	: transcription termination/antitermination L factor
rpoE	: RNA polymerase, sigma 24 (sigma E) factor

Translation
miaA	: delta(2)-isopentenylpyrophosphate tRNA-adenosine transferase
pcnB	: poly(A) polymerase I
pheT	: phenylalanine tRNA synthetase, beta subunit
proS	: prolyl-tRNA synthetase
rnpA	: protein C5 component of RNase P
rplJ	: 50S ribosomal subunit protein L10
rpsF	: 30S ribosomal subunit protein S6

Folding, Sorting and Degradation
glnD	: uridylyltransferase
lepB	: leader peptidase (signal peptidase I)
sspA	: stringent starvation protein A
tig	: peptidyl-prolyl cis/trans isomerase (trigger factor)
ybbN	: predicted thioredoxin domain-containing protein

Replication and Repair
dinG	: ATP-dependent DNA helicase
dnaQ	: DNA polymerase III epsilon subunit
dnaX	: DNA polymerase III/DNA elongation factor III, tau and gamma subunits
hepA	: RNA polymerase-associated helicase protein (ATPase and RNA polymerase recycling factor)
holA	: DNA polymerase III, delta subunit
holB	: DNA polymerase III, delta prime subunit
mutL	: methyl-directed mismatch repair protein
recG	: ATP-dependent DNA helicase
recN	: recombination and repair protein
recO	: gap repair protein
ruvA	: component of RuvABC resolvasome, regulatory subunit
sbcB	: exonuclease I
seqA	: regulatory protein for replication initiation
uvrD	: DNA-dependent ATPase I and helicase II

Membrane Transport
msbA	: fused lipid transporter subunits of ABC superfamily: membrane component/ATP-binding component
uraA	: uracil transporter
ybeX	: predicteed ion transport
ynfH	: oxidoreductase, membrane subunit

Signal Transduction
arcA	: DNA-binding response regulator in two-component regulatory system with ArcB or CpxA
cpxA	: sensory histidine kinase in two-component regulatory system with CpxR
glnD	: uridylyltransferase
phoR	: sensory histidine kinase in two-component regulatory system with PhoB
yfhK	: predicted sensory kinase in two-component system

Cell growth and Death
ftsB	: cell division protein
ftsI	: transpeptidase involved in septal peptidoglycan synthesis (penicillin-binding protein 3)
ftsQ	: membrane anchored protein involved in growth of wall at septum
mrdB	: cell wall shape-determining protein
mreC	: cell wall structural complex MreBCD transmembrane component MreC
mreD	: cell wall structural complex MreBCD transmembrane component MreD
zipA	: cell division protein involved in Z ring assembly

Unclassified Proteins
apbA	:	
cvpA	: membrane protein required for colicin V production
cysB	: DNA-binding transcriptional dual regulator, O-acetyl-L-serine-binding
dacA	: D-alanyl-D-alanine carboxypeptidase (penicillin-binding protein 5)
dsbC	: protein disulfide isomerase II
dsbD	:
era	: membrane-associated, 16S rRNA-binding GTPase
fis	: global DNA-binding transcriptional dual regulator
gcvR	: DNA-binding transcriptional repressor, regulatory protein accessory to GcvA
hlpA	:
imp	: exported protein required for envelope biosynthesis and integrity
lnt	: apolipoprotein N-acyltransferase
lolA	: chaperone for lipoproteins
mltB	: membrane-bound lytic murein transglycosylase B
mrcA	: fused penicillin-binding protein 1a: murein transglycosylase/murein transpeptidase
mrdA	: transpeptidase involved in peptidoglycan synthesis (penicillin-binding protein 2)
nlpD	: predicted outer membrane lipoprotein
pbpG	: D-alanyl-D-alanine endopeptidase
rbn	: binuclear zinc phosphodiesterase
rlpB	: minor lipoprotein
rseA	: anti-sigma factor
rseB	: anti-sigma factor
slp	: outer membrane lipoprotein
sspB	: ClpXP protease specificity-enhancing factor
surA	: peptidyl-prolyl cis-trans isomerase (PPIase)
wrbA	: predicted flavoprotein in Trp regulation
yabB	:
yaeL	:
yafS	: predicted S-adenosyl-L-methionine-dependent methyltransferase
ybiS	: hypothetical protein
ycaI	: conserved inner membrane protein
ycfC	:
yciM	: hypothetical protein
yeaZ	: predicted peptidase
yecG	:
yejK	: nucleotide associated protein
yfcB	:
yfcN	: hypothetical protein
yfgC	: predicted peptidase
yfgD	: predicted oxidoreductase
yfgM	: hypothetical protein
ygbO	:
ygfY	: hypothetical protein
ygfZ	: predicted folate-dependent regulatory protein
yhbN	:
yhdP	: conserved membrane protein, predicted transporter
yibP	:
yjaG	: hypothetical protein
yjeE	: ATPase with strong ADP affinity
yjgD	:
yjgP	: conserved inner membrane protein
ypjD	: predicted inner membrane protein
yqcC	: hypothetical protein
yqiC	: hypothetical protein
yraM	: hypothetical protein
ytfM	: predicted outer membrane protein and surface antigen
ytfN	: hypothetical protein
