# Supplementary material for: Dynamic Changes in Protein Functional Linkage Networks Revealed by Integration with Gene Expression Data
Source: PLoS Comput Biol. 2008 Nov 28;4(11):e1000237. doi: 10.1371/journal.pcbi.1000237 (PMC2580820; doi:10.1371/journal.pcbi.1000237)
Supplement: Table S4 — Functions of the high centrality measure nodes in the comparison set UWT-TWT and UML-TML. (0.07 MB RTF) [file pcbi.1000237.s004.rtf]

Supplementary Table S4: Functions of the high centrality measure nodes in the comparison set UWT-TWT and UML-TML.
UWT-TWT
Functions of the critical proteins of UWT
amn	AMP nucleosidase
avtA	valine-pyruvate aminotransferase
betT	choline transporter of high affinity
bglX	beta-D-glucoside glucohydrolase, periplasmic
cydA	cytochrome d terminal oxidase, subunit I
cydB	cytochrome d terminal oxidase, subunit II
deoR	DNA-binding transcriptional repressor
dld	D-lactate dehydrogenase, FAD-binding, NADH independent
dnaK	chaperone Hsp70, co-chaperone with DnaJ
dppD	dipeptide transporter
dut	deoxyuridinetriphosphatase
fsr	predicted fosmidomycin efflux system
gatD	galactitol-1-phosphate dehydrogenase, Zn-dependent and NAD(P)-binding
glgA	glycogen synthase
gltJ	glutamate and aspartate transporter subunit
kdtA	3-deoxy-D-manno-octulosonic-acid transferase 
lldP	L-lactate permease
mltB	membrane-bound lytic murein transglycosylase B
nrdE	ribonucleoside-diphosphate reductase 2, alpha subunit
paaX	DNA-binding transcriptional repressor of phenylacetic acid degradation, aryl-CoA responsive
pdhR	DNA-binding transcriptional dual regulator
phoB	DNA-binding response regulator in two-component regulatory system with PhoR (or CreC)
potB	polyamine transporter subunit
rfaK	lipopolysaccharide core biosynthesis 
rfaQ	lipopolysaccharide core biosynthesis protein
rfaY	lipopolysaccharide core biosynthesis protein
rhsC	rhsC element core protein RshC
sbmA	predicted transporter
speA	biosynthetic arginine decarboxylase, PLP-binding
sugE	multidrug efflux system protein
tehA	potassium-tellurite ethidium and proflavin transporter
trmD	tRNA (guanine-1-)-methyltransferase
tufA	protein chain elongation factor EF-Tu (duplicate of tufB)
uup	fused predicted transporter subunits of ABC superfamily: ATP-binding components
yaaA	hypothetical protein
yafC	predicted DNA-binding transcriptional regulator
yaiW	predicted DNA-binding transcriptional regulator
ybdK	gamma-glutamyl:cysteine ligase
ycgM	predicted isomerase/hydrolase
yciF	hypothetical protein
ydcN	predicted DNA-binding transcriptional regulator
yddW	predicted liprotein
yedJ	predicted phosphohydrolase
yehW	predicted transporter subunit: membrane component of ABC superfamily
yejH	predicted ATP-dependet helicase
yfaL	adhesin
yggX	protein that protects iron-sulfur proteins against oxidative damage
yidB	hypothetical protein
yjjK	fused predicted transporter subunits of ABC superfamily: ATP-binding components
yneH	predicted glutaminase

Functions of the critical proteins of TWT
ampE	predicted inner membrane protein
apt	adenine phosphoribosyltransferase
cobT	nicotinate-nucleotide dimethylbenzimidazole-P phophoribosyl transferase
cysP	thiosulfate transporter subunit
dsbd	thiol:disulfide interchange protein DsbD 
fabD	malonyl-CoA-[acyl-carrier-protein] transacylase
feoB	fused ferrous iron transporter, protein B: GTP-binding protein/membrane protein
fic	stationary-phase protein, cell division
glnD	uridylyltransferase
gsk	inosine/guanosine kinase
guaC	GMP reductase
hemD	uroporphyrinogen III synthase
hemX	predicted uroporphyrinogen III methylase
hemY	predicted protoheme IX synthesis protein
hepA	RNA polymerase-associated helicase protein (ATPase and RNA polymerase recycling factor)
holC	DNA polymerase III, chi subunit
htrb	Lipid A biosynthesis lauroyl acyltransferase 
lnt	apolipoprotein N-acyltransferase
lolA	chaperone for lipoproteins
miaA	delta(2)-isopentenylpyrophosphate tRNA-adenosine transferase
mltB	membrane-bound lytic murein transglycosylase B
mntR	DNA-binding transcriptional regulator of mntH
nfnb	oxygen-insensitive NAD(P)H nitroreductase 
nusA	transcription termination/antitermination L factor
pcnB	poly(A) polymerase I
pheT	phenylalanine tRNA synthetase, beta subunit
plsB	glycerol-3-phosphate O-acyltransferase
pyrC	dihydro-orotase
recN	recombination and repair protein
recO	gap repair protein
rfak	lipopolysaccharide core biosynthesis 
rimI	acetylase for 30S ribosomal subunit protein S18
rseA	anti-sigma factor
rseB	anti-sigma factor
slyB	outer membrane lipoprotein
slyX	hypothetical protein
speG	spermidine N1-acetyltransferase
surA	peptidyl-prolyl cis-trans isomerase (PPIase)
surE	broad specificity 5'(3')-nucleotidase and polyphosphatase
tag	3-methyl-adenine DNA glycosylase I, constitutive
tdh	threonine 3-dehydrogenase, NAD(P)-binding
tmk	thymidylate kinase 
ugpC	glycerol-3-phosphate transporter subunit
yaeL	--
ybbN	predicted thioredoxin domain-containing protein
ybhN	conserved inner membrane protein
ycfC	hypothetical protein YcfC 
ychN	hypothetical protein
yebR	hypothetical protein
yedA	predicted inner membrane protein
yedU	
yejK	nucleotide associated protein
yejM	predicted hydrolase, inner membrane
yfcN	hypothetical protein
yfgC	predicted peptidase
ygfZ	predicted folate-dependent regulatory protein
yhdP	conserved membrane protein, predicted transporter
yhfK	conserved inner membrane protein
yhgF	predicted transcriptional accessory protein
yicC	hypothetical protein
yieF	chromate reductase, Class I, flavoprotein
yigA	hypothetical protein
yjaG	hypothetical protein
yjgP	conserved inner membrane protein
yjgR	predicted ATPase
yneI	predicted aldehyde dehydrogenase
yqcC	hypothetical protein
yraM	hypothetical protein
ytfN	hypothetical protein
	
UML-TML

Functions of the critical proteins of UML
ampC	beta-lactamase/D-alanine carboxypeptidase
appC	cytochrome bd-II oxidase, subunit I
bax	hypothetical protein
fdoG	formate dehydrogenase-O, large subunit
fic	stationary-phase protein, cell division
gatA	galactitol-specific enzyme IIA component of PTS
kdpE	DNA-binding response regulator in two-component regulatory system with KdpD
kdtA	3-deoxy-D-manno-octulosonic-acid transferase 
murF	UDP-N-acetylmuramoyl-tripeptide:D-alanyl-D-alanine ligase
nikD	nickel transporter subunit
nmpC	outer membrane porin NmpC 
rho	transcription termination factor
ribF	bifunctional riboflavin kinase/FAD synthetase
rlpB	minor lipoprotein
rpsK	30S ribosomal subunit protein S11
slt	lytic murein transglycosylase, soluble
speG	spermidine N1-acetyltransferase
surE	broad specificity 5'(3')-nucleotidase and polyphosphatase
tas	predicted oxidoreductase, NADP(H)-dependent aldo-keto reductase
tufA	protein chain elongation factor EF-Tu (duplicate of tufB)
tyrA	fused chorismate mutase T/prephenate dehydrogenase
uxaA	altronate hydrolase
uxaB	altronate oxidoreductase, NAD-dependent
yajG	predicted lipoprotein
ybaZ	predicted methyltransferase
ycdY	hypothetical protein
yddG	predicted methyl viologen efflux pump
ydhK	conserved inner membrane protein
yedU	 
yfiM	hypothetical protein
yggD	predicted DNA-binding transcriptional regulator
yhbE	conserved inner membrane protein
yhdW	predicted amino-acid transporter subunit
yhjE	predicted transporter
yihL	predicted DNA-binding transcriptional regulator
yjbN	
yqaA	conserved inner membrane protein
ytfG	NAD(P)H:quinone oxidoreductase


Functions of the critical proteins of TML
agaA	predicted truncated N-acetylgalactosamine-6-phosphate deacetylase 
agaR	DNA-binding transcriptional dual regulator
appY	DLP12 prophage; DNA-binding transcriptional activator
argG	argininosuccinate synthetase
aspS	aspartyl-tRNA synthetase
cvpA	membrane protein required for colicin V production
cysB	DNA-binding transcriptional dual regulator, O-acetyl-L-serine-binding
ddg	palmitoleoyl-acyl carrier protein (ACP)-dependent acyltransferase 
dedA	conserved inner membrane protein
dnaA	chromosomal replication initiator protein DnaA, DNA-binding transcriptional dual regulator
dnaE	DNA polymerase III alpha subunit
era	membrane-associated, 16S rRNA-binding GTPase
folC	bifunctional folylpolyglutamate synthase/ dihydrofolate synthase
ftsQ	membrane anchored protein involved in growth of wall at septum
gcvA	DNA-binding transcriptional dual regulator
glgX	glycogen debranching enzyme
gltJ	glutamate and aspartate transporter subunit
ilvM	acetolactate synthase II, small subunit
katE	hydroperoxidase HPII(III) (catalase)
kdta	3-deoxy-D-manno-octulosonic-acid transferase 
lepB	leader peptidase (signal peptidase I)
lgt	phosphatidylglycerol-prolipoprotein diacylglyceryl transferase
lpxB	tetraacyldisaccharide-1-P synthase
lpxC	UDP-3-O-acyl N-acetylglucosamine deacetylase
lspA	prolipoprotein signal peptidase (signal peptidase II)
mfd	transcription-repair coupling factor
mhpF	acetaldehyde-CoA dehydrogenase II, NAD-binding
miaA	delta(2)-isopentenylpyrophosphate tRNA-adenosine transferase
mrdB	cell wall shape-determining protein
murD	UDP-N-acetylmuramoyl-L-alanine:D-glutamate ligase
murE	UDP-N-acetylmuramoyl-L-alanyl-D-glutamate:meso-diaminopimelate ligase
narX	sensory histidine kinase in two-component regulatory system with NarL
nusA	transcription termination/antitermination L factor
paaE	predicted multicomponent oxygenase/reductase subunit for phenylacetic acid degradation
pal	peptidoglycan-associated outer membrane lipoprotein
pcnB	poly(A) polymerase I
pdxA	4-hydroxy-L-threonine phosphate dehydrogenase, NAD-dependent
pepA	aminopeptidase A, a cyteinylglycinase
pheT	phenylalanine tRNA synthetase, beta subunit
pmrD	polymyxin resistance protein B
ppsA	phosphoenolpyruvate synthase 
prlA	preprotein translocase membrane subunit 
recJ	ssDNA exonuclease, 5' --> 3'-specific
rph	defective ribonuclease PH
rplD	50S ribosomal subunit protein L4
rplQ	50S ribosomal subunit protein L17
rpoA	RNA polymerase, alpha subunit
rpoE	RNA polymerase, sigma 24 (sigma E) factor
rpoH	RNA polymerase, sigma 32 (sigma H) factor
rpoZ	RNA polymerase, omega subunit
rpsA	30S ribosomal subunit protein S1
rpsD	30S ribosomal subunit protein S4
rpsF	30S ribosomal subunit protein S6
sbcB	exonuclease I
secD	SecYEG protein translocase auxillary subunit
spoT	bifunctional (p)ppGpp synthetase II/ guanosine-3',5'-bis pyrophosphate 3'-pyrophosphohydrolase
srlD	sorbitol-6-phosphate dehydrogenase
sspA	stringent starvation protein A
sspB	ClpXP protease specificity-enhancing factor
sugE	multidrug efflux system protein
tas	predicted oxidoreductase, NADP(H)-dependent aldo-keto reductase
tig	peptidyl-prolyl cis/trans isomerase (trigger factor)
tmk	thymidylate kinase 
tolB	periplasmic protein
tolQ	membrane spanning protein in TolA-TolQ-TolR complex
yaaH	conserved inner membrane protein associated with acetate transport
yaeL	hypothetical protein
yaeT	hypothetical protein
yafP	predicted acyltransferase with acyl-CoA N-acyltransferase domain
yaiE	hypothetical protein
ybbL	predicted transporter subunit: ATP-binding component of ABC superfamily
ydhI	predicted inner membrane protein
yfiO	predicted lipoprotein
ygcJ	hypothetical protein
ygdP	nucleotide hydrolase 
ygfZ	predicted folate-dependent regulatory protein
yggS	predicted enzyme
yhaJ	predicted DNA-binding transcriptional regulator
yhdT	conserved inner membrane protein
yhiN	predicted oxidoreductase with FAD/NAD(P)-binding domain
yi21_4	--
yjeE	ATPase with strong ADP affinity
yjeQ	hypothetical protein
ymfK	e14 prophage; repressor protein phage e14
yojI	fused predicted multidrug transport subunits of ABC superfamily: membrane component/ATP-binding component
yraL	predicted methyltransferase
