# Supplementary figures and images for: Dynamic Changes in Protein Functional Linkage Networks Revealed by Integration with Gene Expression Data
Source: PLoS Comput Biol. 2008 Nov 28;4(11):e1000237. doi: 10.1371/journal.pcbi.1000237 (PMC2580820; doi:10.1371/journal.pcbi.1000237)

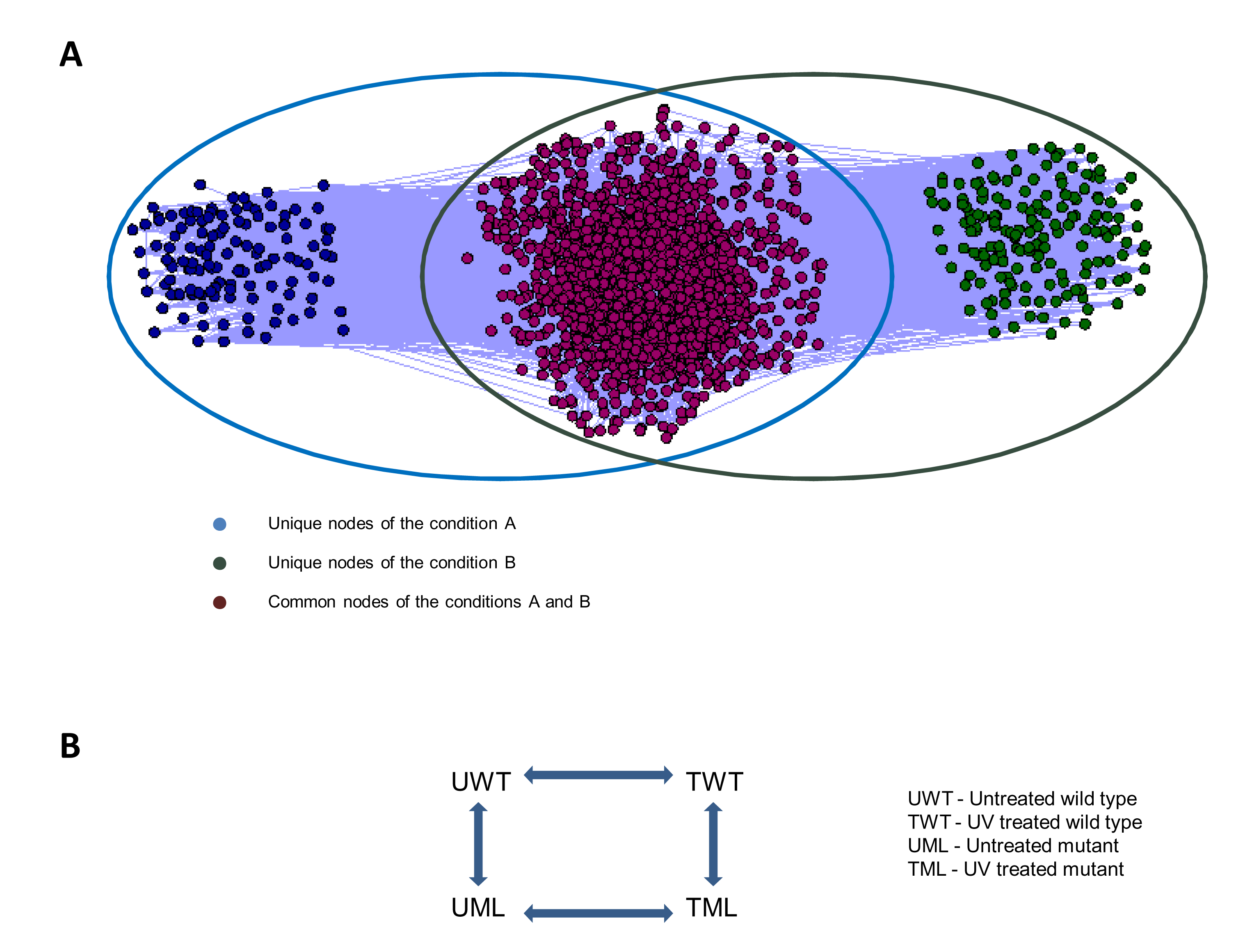

Supplement: Figure S1 — Differential gene expression and comparison of networks. (A) Pictorial representation of differential gene expression in the network context. Red and green represent the nodes expressed uniquely under the defined conditions, whereas blue nodes are expressed under both the conditions. (B) Four-way comparison of the networks. UWT, wild type; TWT, UV treated wild type; UML, lexA mutant; TML, UV-treated lexA mutant. (0.47 MB TIF) [file pcbi.1000237.s006.tif]

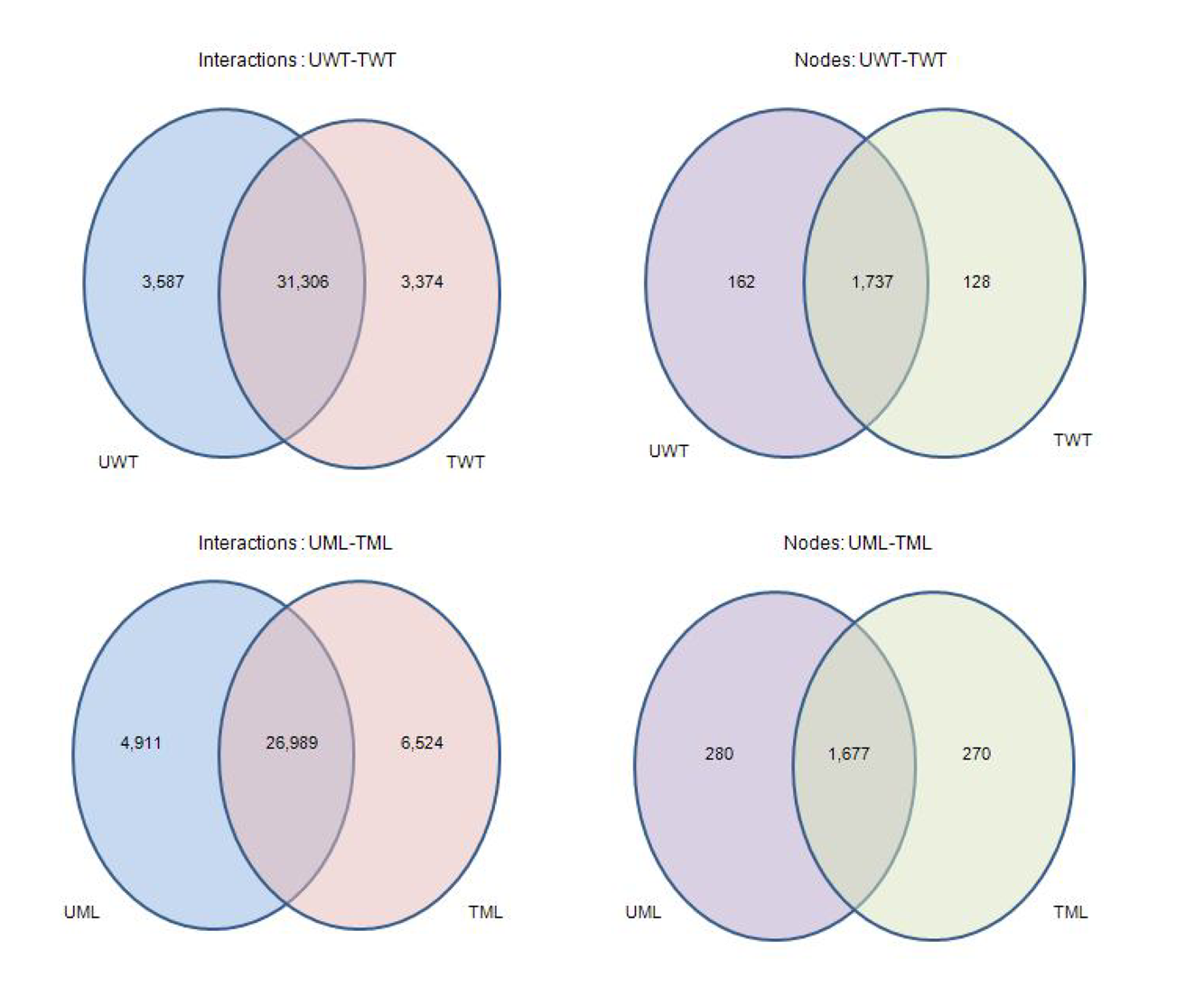

Supplement: Figure S2 — The overlap of the interactions and the nodes in UWT-TWT and UML-TML. (0.76 MB TIF) [file pcbi.1000237.s007.tif]

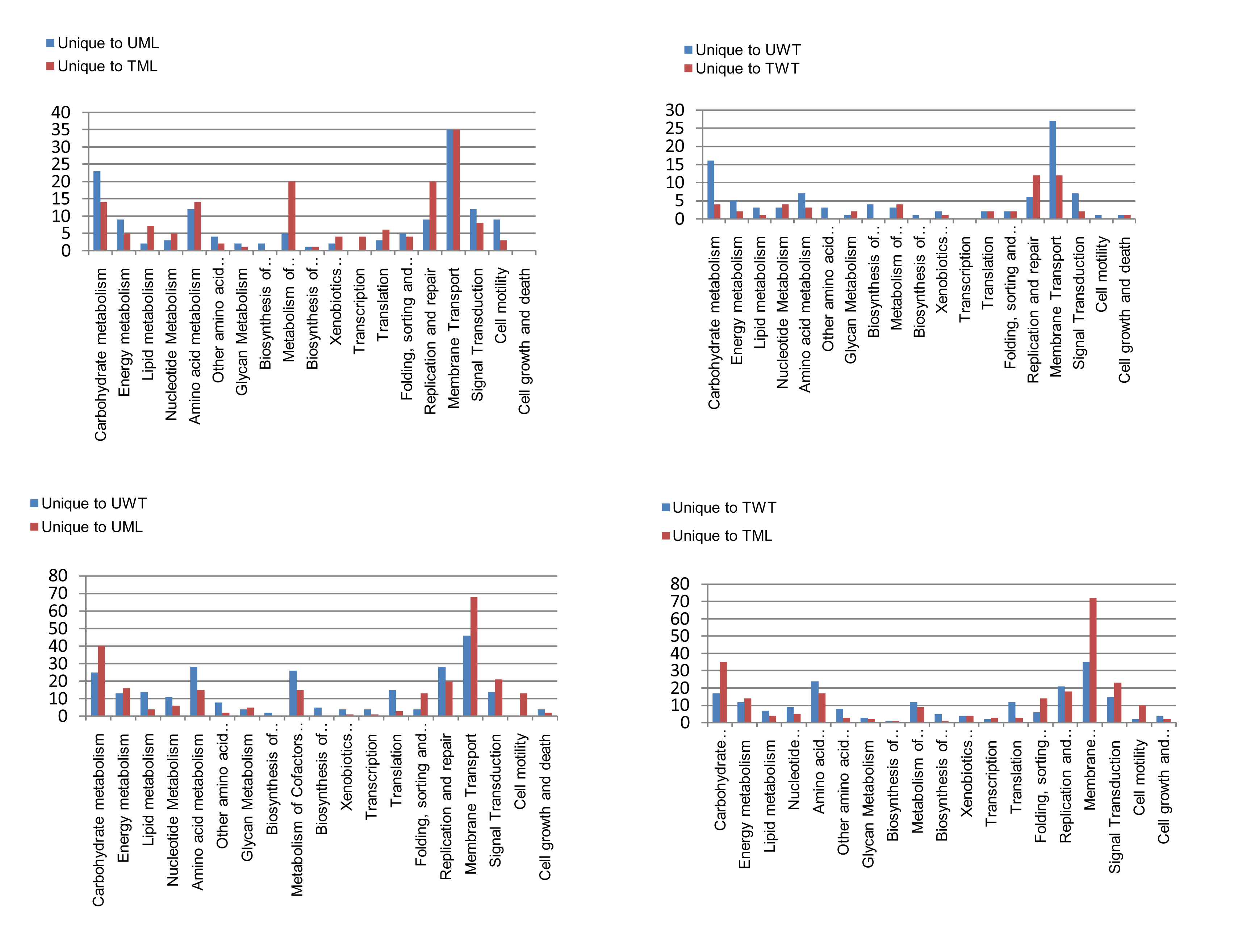

Supplement: Figure S3 — Mapping of unique nodes to different metabolic pathways. (0.45 MB TIF) [file pcbi.1000237.s008.tif]

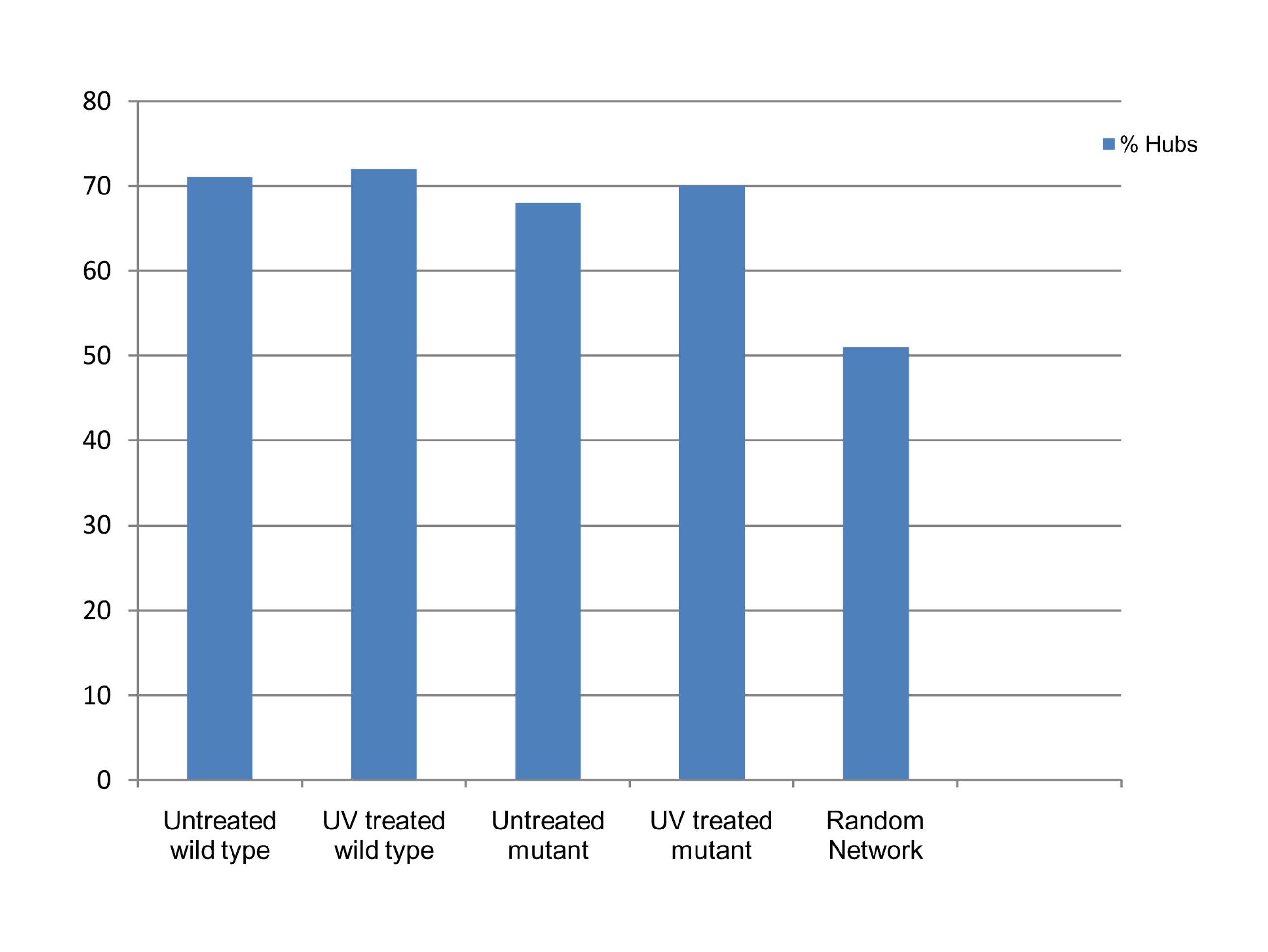

Supplement: Figure S4 — Expression of the hubs. (0.23 MB TIF) [file pcbi.1000237.s009.tif]
